# An Isothermal Deoxyribozyme Sensor for Rapid Detection of Enteroviral RNA

**DOI:** 10.3390/bios15090562

**Published:** 2025-08-27

**Authors:** Begüm Şaş, Anastasiia Dmitrievna Kirichenko, Marina Anatolyevna Kapitonova, Anna Vyacheslavovna Shabalina, Olga Ilyinichna Kanaeva, Tamer Mohammed El-Messery, Vladimir Georgievich Dedkov, Anna Sergeevna Dolgova

**Affiliations:** 1 Laboratory of Pathogen Molecular Genetics, Saint Petersburg Pasteur Institute, 197101 St. Petersburg, Russia; anastasiiakirichenko97@gmail.com (A.D.K.); kapitonova.marin@gmail.com (M.A.K.); shabalina.anna.v@gmail.com (A.V.S.); vgdedkov@yandex.ru (V.G.D.); annadolgova@inbox.ru (A.S.D.); 2 Faculty of Biotechnologies, ITMO University, 191002 St. Petersburg, Russia; tmelmessery@yahoo.com; 3 Laboratory of Etiology and Control of Viral Infections, Saint Petersburg Pasteur Institute, 197101 St. Petersburg, Russia; ol.kanaeva@gmail.com; 4 Martsinovsky Institute of Medical Parasitology, Tropical and Vector Borne Diseases, Sechenov First Moscow State Medical University (Sechenov University), 119048 Moscow, Russia

**Keywords:** enteroviral RNA detection, environmental water matrices, isothermal biosensor, NASBA-assisted amplification, split DNAzyme

## Abstract

Enteric viruses are a major cause of waterborne infections due to their high environmental stability and extremely low infectious dose. Current molecular diagnostic methods, while accurate, often depend on thermal cycling and centralized laboratory facilities, limiting their applicability in decentralized or resource-limited settings. In this study, we developed an isothermal biosensor based on a split deoxyribozyme that reconstitutes its catalytic core upon hybridization with a conserved sequence of enteroviral RNA. This activation leads to site-specific cleavage of a fluorogenic substrate, producing a quantifiable fluorescent signal. The system was experimentally validated using both synthetic enteroviral RNA and RNA extracted from environmental water samples. To enhance detection sensitivity, the DNAzyme-based assay was coupled with isothermal RNA amplification. The results demonstrate high selectivity and compatibility with real-world samples, supporting the sensor’s utility for field-deployable viral RNA detection. Overall, this study highlights the potential of the DNAzyme-based platform as a portable, sequence-specific, and amplification-assisted diagnostic tool for environmental surveillance of enteric viruses.

## 1. Introduction

Foodborne and waterborne viruses remain a persistent global health concern, particularly in regions with poorly developed sanitation infrastructure [1]. Enteric viruses such as norovirus, rotavirus, and members of the *Enterovirus* genus are especially problematic due to their high prevalence, remarkable environmental stability, and minimal infective dose [2,3]. In this study, we target a conserved RNA region shared by several environmentally relevant human enteroviruses, including Coxsackievirus B4, Coxsackievirus B5, Echovirus 3, Echovirus 11, and Echovirus 13, which are commonly detected in environmental water samples. As non-enveloped RNA viruses, they remain infectious in complex matrices such as food and water even after exposure to adverse conditions, including disinfectants, low pH, and freezing temperatures [3,4]. Numerous outbreaks linked to contaminated produce, shellfish, and inadequately treated drinking water underscore the urgent need for accessible and decentralized viral monitoring tools [5].

Current molecular diagnostic approaches, such as reverse transcription polymerase chain reaction (RT-PCR) and quantitative PCR (qPCR), provide high sensitivity but require thermal cycling, specialized instrumentation, and trained personnel [6]. Isothermal techniques like loop-mediated isothermal amplification (LAMP) have improved portability. However, their reliance on enzymatic reactions and complex readouts still limits rapid field deployment [7,8].

Biosensor technologies, particularly those using nucleic acid recognition elements, are promising for rapid viral detection in diverse matrices [9,10,11]. Catalytic DNA-based biosensors (*Deoxyribozymes*, DNAzymes) offer key advantages. They are synthetically accessible, thermodynamically stable, and capable of site-specific RNA cleavage under physiological conditions [9,12]. The 10–23 DNAzyme motif, in particular, is well-characterized for its sequence-dependent catalytic activity [10,13].

In this work, a split 10–23 DNAzyme sensor was combined with nucleic acid sequence-based amplification (NASBA) to allow ultrasensitive and selective detection of enteroviral RNA under isothermal conditions. The catalytic unit forms only upon hybridization with a conserved enterovirus sequence, triggering fluorophore–quencher substrate cleavage and producing a signal. Performance was confirmed using synthetic, in vitro transcribed, and environmentally derived viral RNA. These results support the deployment of amplification-assisted DNAzyme biosensors for decentralized monitoring of enteric viruses in aquatic environments.

## 2. Materials and Methods

### 2.1. Materials

All oligonucleotides used in this study, including the split DNAzyme strands (Dz1, Dz2) and the fluorogenic substrate (Fsub) labelled with 5′6-carboxyfluorescein (FAM) and 3′ Black Hole Quencher-1 (BHQ1), were synthesized locally (DNK-Sintez, Moscow, Russia). The complete list of sequences is provided in Tables S1 and S2. Analytical-grade chemicals, including potassium chloride (KCl), calcium chloride (CaCl_2_), sodium hydroxide (NaOH), and Tris-HCl, were purchased from PanReach AppliChem (Chicago, IL, USA). RNase-/DNase-free ultrapure water (Thermo Fisher Scientific, Waltham, MA, USA) was used in all experiments to preserve RNA integrity. In vitro transcription of target RNA was performed using the T7 RiboMAX™ Express Large Scale RNA Production System (Promega Corporation, Madison, WI, USA) followed by DNase I treatment (Promega Corporation, Madison, WI, USA) to remove DNA templates. Reverse transcription was carried out with the RevertAid First Strand cDNA Synthesis Kit (Thermo Fisher Scientific, Waltham, MA, USA). NASBA amplification was performed using a commercial kit from AMSBIO (Cambridge, MA, USA) according to manufacturer instructions. RNA quality was evaluated using a NanoDrop One spectrophotometer (Thermo Fisher Scientific, Waltham, MA, USA). Only samples with acceptable A260/A280 ratios were included in assays. AMPure XP magnetic beads (Beckman Coulter, Brea, CA, USA) were used for nucleic acid purification. RNA from viral samples was extracted using the RIBO-prep Nucleic Acid Isolation Kit (AmpliSens, Moscow, Russian Federation). All reagents and consumables were RNase-/DNase-free to prevent nucleic acid degradation or contamination. Fluorescence measurements were obtained using a T16-ISO Isothermal Fluorescence Reader (Axxin, Eaglemont, VIC, Australia).

### 2.2. Bioinformatics Analysis

Whole genome sequences of *Enterovirus* strains previously isolated from environmental water and foodborne samples were retrieved from the GenBank database (NCBI) [14]. Highly conserved regions suitable for DNAzyme-based biosensor targeting were identified via multiple sequence alignment in MEGA version 11 [15]. Conserved sequences were then analyzed with the NUPACK web platform to assess potential secondary structures that could hinder DNAzyme hybridization or catalytic activity [16]. Based on these results, a 178-nucleotide conserved segment (GenBank accession number PP558478.1, region 350–475) was selected for biosensor targeting and amplification workflows.

### 2.3. Positive Control Construction

To obtain a reliable RNA source for assay development, the selected region was cloned into a plasmid under the control of a T7 promoter. This recombinant construct served as a positive control template for in vitro transcription. The plasmid was linearized and transcribed using a T7-based transcription kit to produce synthetic RNA targets. The oligonucleotide sequences used for this assembly are listed in Table S1.

### 2.4. Deoxyribozyme-Based Biosensor Assay

Fluorescence detection in the DNAzyme-based biosensor system relied on substrate cleavage initiated by catalytic core reconstitution upon hybridization with the target RNA. The fluorogenic substrate was labelled with FAM at the 5′ end and BHQ1 at the 3′ end, enabling signal generation upon cleavage. Each reaction, with a final volume of 50 µL, contained 1 µL of a DNAzyme strand mixture (Dz1 and Dz2, 100 nM each), 0.5 µL of Fsub (100 nM), 0.5 µL of target RNA (100 nM), the optimized concentration of Ca^2+^ ions, and the appropriate reaction buffer. In negative controls, target RNA was replaced with an equivalent volume of nuclease-free water. All preparations were performed in PCR tubes on ice to preserve RNA integrity, and pipetting steps were conducted under cold conditions. Samples were briefly vortexed to ensure homogeneity. Fluorescence measurements were carried out using an Axxin T16-ISO isothermal fluorometer (Axxin, Eaglemont, VIC, Australia), with incubation at 41 °C for 20 min [17]. The excitation and emission wavelengths for FAM were set to 495 nm and 517 nm, respectively. Each experiment was performed in triplicate. The mean fluorescence intensity of negative control reactions was recorded as F_0_, whereas that of target RNA-containing reactions was denoted F_1_. Signal enhancement was expressed as the F_1_/F_0_ ratio, which reflects the extent of substrate cleavage upon DNAzyme activation [18,19]. Samples with an F_1_/F_0_ ratio greater than 1.5 were considered positive, while those with values equal to or below this threshold were considered negative.

### 2.5. Reaction Buffers and Cofactor Optimization

The catalytic efficiency of the DNAzyme-based biosensor was evaluated by testing four reaction buffers (RB1–RB4) and their additive-enriched variants (RB1*–RB4*) [20]. The composition and final concentration of each buffer are provided in Table S3. Chemically modified buffers contained 0.06% Triton X-100 and 0.05% dimethyl sulfoxide (DMSO). Triton X-100 improved oligonucleotide dispersion by reducing surface tension. DMSO disrupted secondary structure formation, thereby enhancing DNAzyme accessibility to RNA targets under isothermal conditions [19,20]. Mixing and measurement procedures were performed as described in the previous section. To maximize catalytic activity, the Ca^2+^ concentration in the reaction mixture was systematically optimized. Calcium chloride (CaCl_2_) was added in varying amounts to achieve final concentrations ranging from 5 mM to 100 mM, as outlined in the optimization series [21,22]. All reactions were adjusted to a final volume of 50 µL using the pre-optimized buffer system and briefly vortexed to ensure homogeneity. The F_1_/F_0_ ratio was used to evaluate the effect of Ca^2+^ concentration on biosensor performance. The condition yielding the highest signal enhancement was defined as the optimal cofactor level for the system.

### 2.6. Determination of Detection and Quantification Limits

The analytical sensitivity of the DNAzyme-based biosensor was determined by recording fluorescence responses to serial dilutions of target RNA [23]. Synthetic RNA was tested across a concentration range of 0.5–100 nM, while in vitro transcribed RNA was assessed from 1–100 nM. Each reaction was prepared in a final volume of 50 µL, incorporating the optimal Ca^2+^ concentration and buffer system identified during optimization. A four-parameter Hill function was applied to model the dose–response trend for visualization purposes only (Equation (1)). The limit of detection (*LOD*) was defined as the lowest RNA concentration distinguishable from the background, and the limit of quantification (*LOQ*) as the minimum concentration allowing reliable quantification [24,25]. Both values were statistically calculated using the blank signal distribution (Equation (2)).

Equation (1)

(1) $$F=A+\frac{D-A}{1+{\left(\frac{C}{\left[T\right]}\right)}^{B}}$$

Equation (2)

(2) $$LOD=\frac{3.3\cdot \sigma }{S},LOQ=\frac{10\cdot \sigma }{S}$$

### 2.7. Selectivity Assessment

The specificity of the DNAzyme-based biosensor was assessed by testing its response to a panel of viral RNAs commonly detected in environmental samples. Alongside the target enterovirus, six non-target viruses were examined: norovirus; rotavirus A; human astrovirus; sapovirus; hepatitis A virus; and adenovirus. Synthetic viral RNAs were tested at a final concentration of 100 nM under identical experimental conditions [19].

### 2.8. Viral Isolation and Nucleic Acid Sequence-Based Amplification (NASBA) Integration

To improve the sensitivity of the DNAzyme-based biosensor for detecting low-abundance viral RNA in environmental samples, a NASBA step was incorporated into the workflow [25,26]. Viruses were isolated from sewage concentrate samples using RD (human rhabdomyosarcoma) cell line (Global Polio Laboratory Network, NIBSC, Hertfordshire, UK) and HEp-2 (human laryngeal carcinoma) cell line (Global Polio Laboratory Network, NIBSC, Hertfordshire, UK), following the WHO protocol [27]. The enterovirus type was identified by partial sequencing of the VP1 genomic region with NPEV-specific primers [28]. Total RNA was extracted and purified using the RIBO-prep Nucleic Acid Isolation Kit (Amplisens, Moscow, Russia). RNA was first reverse transcribed into complementary DNA (cDNA) with the RevertAid First Strand cDNA Synthesis Kit (Thermo Fisher Scientific, Waltham, MA, USA). The cDNA was then amplified via polymerase chain reaction (PCR) using specific primers. The forward primer included a T7 promoter sequence, as described previously [26]. The resulting double-stranded DNA (dsDNA) served as a template for NASBA. RNA amplification was performed using a commercial NASBA kit (AMSBIO, Cambridge, MA, USA) in accordance with manufacturer instructions. Upon completion of the NASBA reaction, 0.5 µL of the amplified RNA product was directly added to the DNAzyme fluorescence reaction mixture without purification.

## 3. Results

### 3.1. Target-Induced Reassembly of Split DNAzyme Biosensor

**Figure 1:**
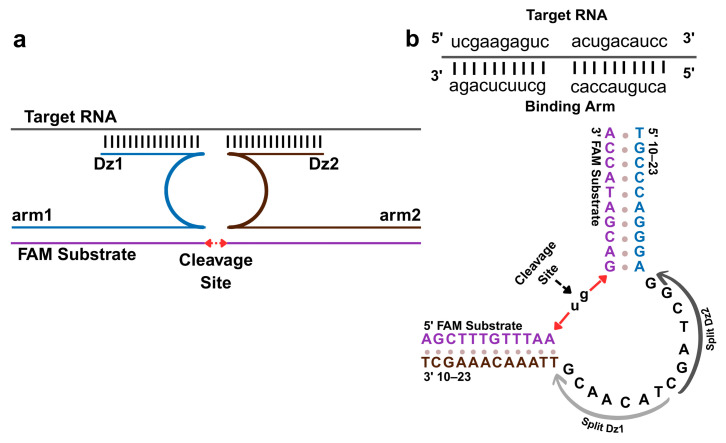
DNAzyme-based detection of enteroviral RNA. (**a**) Schematic of target-induced DNAzyme reassembly via Dz1 and Dz2 hybridization with conserved regions of the target enteroviral RNA, restoring the catalytic core of the 10–23 DNAzyme. This reassembly triggers cleavage of a fluorogenic hairpin substrate (Fsub) positioned between a 5′-FAM fluorophore and a 3′-BHQ1 quencher, generating a fluorescent signal. (**b**) Molecular representation of the 10–23 catalytic core. Upon target-induced activation, site-specific cleavage occurs at the canonical u-g site within the FAM substrate, resulting in separation of the fluorophore and quencher for fluorescence detection.

A split DNAzyme system was developed for sequence-specific recognition of enteroviral RNA. The design consists of two oligonucleotide strands, Dz1 and Dz2, each containing partial sequences of the 10–23 DNAzyme motif. Upon binding to adjacent regions of the target RNA, the two strands reassemble into a catalytically active complex that cleaves a fluorophore-quencher hairpin substrate labelled with 5′-FAM and 3′-BHQ1. It is shown schematically in Figure 1. Cleavage separates the fluorophore from the quencher, generating a fluorescent signal proportional to the concentration of target RNA. The binding arms were designed through bioinformatics analysis of conserved enteroviral sequences, ensuring high sequence fidelity and minimizing off-target interactions. In the absence of the target, fluorescence remains at background levels, indicating strict conditional activation. This hybridization-induced sensing mechanism supports high analytical specificity and minimal background noise, making it suitable for quantitative RNA detection.

### 3.2. Buffer Optimization Enhances DNAzyme Catalytic Efficiency

**Figure 2:**
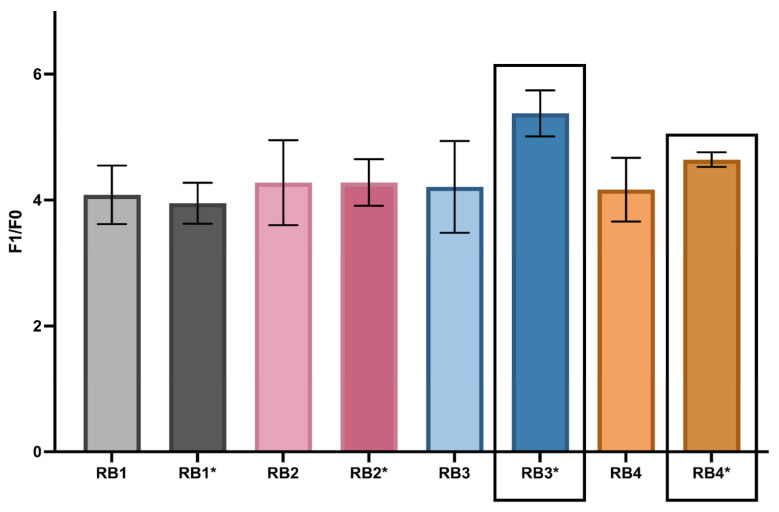
Buffer-dependent modulation of DNAzyme biosensor performance. F_1_/F_0_ fluorescence ratios measured across four buffers (RB1-RB4) and their additive-enriched variants (RB1*–RB4*), each supplemented with Triton X-100 and DMSO, are shown. RB3* and RB4* showed significantly higher catalytic activity compared to other formulations. Data are presented as mean ± s.d. from three independent replicates.

To optimize the catalytic performance of the biosensor, multiple buffer systems were systematically evaluated. Four base buffers (RB1–RB4) and their additive-enriched versions (RB1*–RB4*) were tested, each containing 0.06% Triton X-100 and 0.05% DMSO. Fluorescence kinetics (Figure 2) were monitored under isothermal conditions using the F_1_/F_0_ ratio, where F_1_ represents the endpoint fluorescence in the presence of synthetic target RNA, and F_0_ corresponds to the no-template control (NTC). Full calculations are provided in the Supporting Materials (Table S4).

Among the tested conditions, RB3* yielded the highest average fluorescence ratio (5.383 ± 0.37), while RB4* demonstrated the lowest coefficient of variation (CV = 0.025), indicating strong signal reproducibility. Statistical analysis confirmed a significant difference among buffer conditions (ANOVA, *p* = 0.029), and post hoc Tukey’s test further established that RB3* was significantly superior to RB1* (*p* = 0.014).

Based on these findings, RB3* and RB4* were selected as the optimal formulations, offering a balance between catalytic signal strength and reproducibility. These optimized buffer conditions were subsequently employed in downstream validation experiments.

### 3.3. Calcium-Dependent Catalytic Enhancement

Divalent metal ions are critical cofactors for DNAzyme catalysis, facilitating proper folding of the catalytic core and stabilizing the transition state during phosphodiester bond cleavage. Calcium ions (Ca^2+^) were chosen due to their compatibility with DNAzyme catalysis and their reduced tendency to trigger nonspecific folding. To optimize catalytic performance, a concentration-dependent assay optimization was conducted using six CaCl_2_ concentrations (5–100 mM) in the RB3* and RB4* buffer systems. Reactions were incubated at 41 °C, and catalytic efficiency was evaluated using the F_1_/F_0_ fluorescence ratio (calculations shown in Table S5).

**Figure 3:**
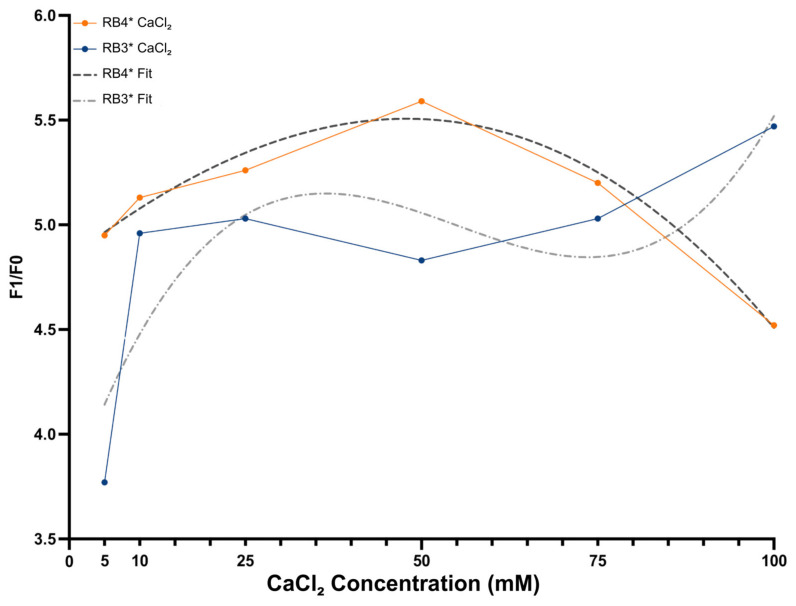
F_1_/F_0_ fluorescence responses measured across different concentration of CaCl_2_ (5–100 mM) in RB3* and RB4* buffer systems. Polynomial regression analysis identified an optimal catalytic response at 48.5 mM in RB4* (R^2^ = 0.960). RB3* showed greater variability and a weaker fit (R^2^ = 0.720). Data represent average values from three independent experiments.

Figure 3 shows that RB4* exhibited a strong, concentration-dependent increase in signal, reaching maximum activation at 48.5 mM Ca^2+^ (R^2^ = 0.960, third-order polynomial fit). In contrast, RB3* showed greater variability, with peak activity at 100 mM Ca^2+^ and lower fit accuracy (R^2^ = 0.720), indicating potential cofactor imbalance or structural instability. Based on signal intensity and reproducibility, 50 mM Ca^2+^ in RB4* was selected as the optimal condition for subsequent assays.

### 3.4. Quantitative Performance of the DNAzyme Biosensor

**Figure 4:**
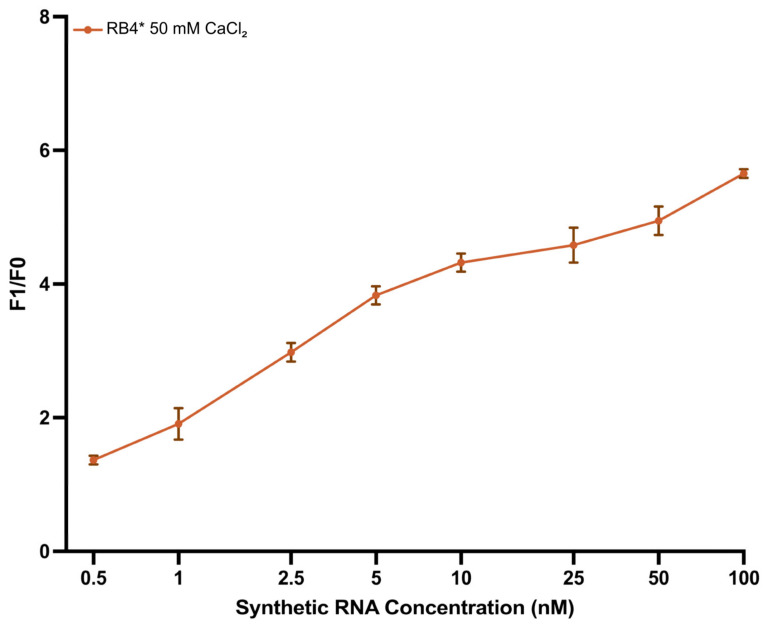
F_1_/F_0_ fluorescence response of the biosensor in RB4 buffer across synthetic RNA concentrations (0.5–100 nM). The data follow a clear sigmoid trend, with F_1_/F_0_ values surpassing 3.0 at ≥2.5 nM RNA. A four-parameter logistic fit yielded a strong correlation (R^2^ = 0.976), indicating high sensitivity and consistency. Data are presented as mean ± s.d. from three replicates. Model fit details are provided in Table S4.

To estimate the quantitative detection capability of the biosensor, synthetic enteroviral RNA was tested across a concentration range of 0.5–100 nM using the optimized RB4* buffer. A clear, concentration-dependent increase in fluorescent signal was observed, with F_1_/F_0_ values exceeding 3.0 at RNA concentrations ≥ 2.5 nM (Figure 4), demonstrating high sensor sensitivity. Sigmoid regression analysis revealed a strong correlation between RNA concentration and signal intensity (R^2^ = 0.976), confirming the robustness of the system across a broad dynamic range.

Based on this model, the theoretical *LOD* and *LOQ* were determined as 0.28 nM and 1.07 nM, respectively. Experimentally, *LOD* and *LOQ* were observed at 0.5 nM and 2.5 nM, respectively (Table S6). Based on this strong performance, RB4* was selected as the optimal condition for all subsequent assays.

### 3.5. Sequence Specificity of the DNAzyme Biosensor

**Figure 5:**
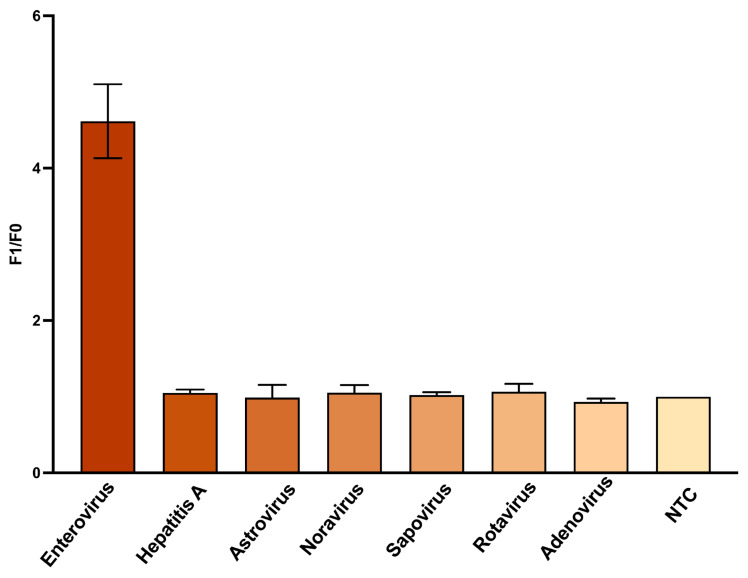
Sequence specificity of the DNAzyme biosensor against a viral RNA panel. F_1_/F_0_ fluorescent signals measured under RB4* buffer conditions show selective activation exclusively by enteroviral RNA. All non-target RNAs produced background-level responses. Data are shown as mean ± s.d. from three replicates.

Precise target recognition is essential for diagnostic platforms designed to detect viral contaminants in complex matrices. To assess the specificity of the split DNAzyme biosensor, a panel of seven viral RNAs was tested, including the synthetic target enterovirus and six non-target species: norovirus, rotavirus A, human astrovirus, sapovirus, hepatitis A virus, and adenovirus. Each RNA sample was tested at 100 nM under optimized conditions. Fluorescence was measured in real-time, and the reported F_1_/F_0_ values are presented in Figure 5 and Table S7. Only enterovirus RNA triggered a strong signal (F_1_/F_0_ = 4.62 ± 0.29), while all non-targets produced background-level responses (F_1_/F_0_ = 0.91 ± 1.21).

This high specificity is a result of the special split design, which requires precise hybridization for catalytic core reassembly. In the absence of sufficient complementarity, the DNAzyme remains inactive. One-way ANOVA confirmed significant discrimination between target and non-targets (*p* < 0.0001), further supporting the platform’s high target sequence accuracy. These findings demonstrate the sensor’s ability to precisely discriminate between target and non-target species.

### 3.6. Sensitivity Analysis Using In Vitro Transcribed Enterovirus RNA 

The biosensor was next tested using a longer RNA target. The RNA was transcribed from a pre-amplified DNA matrix by T7 polymerase. This approach, using targets with a length greater than the hybridization area, ensured the preservation of native secondary structures, providing a more realistic test of biosensor functionality.

**Figure 6:**
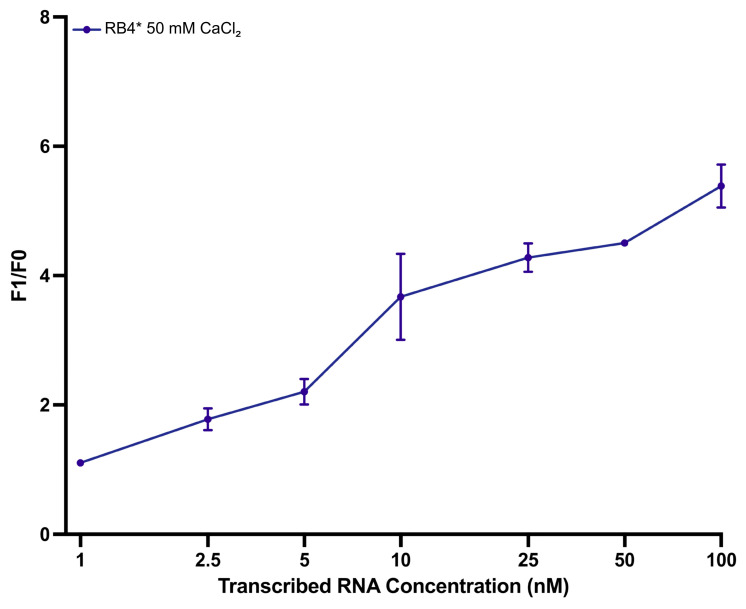
Sensitivity analysis of the DNAzyme biosensor using in vitro transcribed enteroviral RNA. A concentration-dependent increase in normalized fluorescent signal (F_1_/F_0_) was observed across the 1–100 nM range. The data were fitted using a four-parameter Hill model with strong correlation (R^2^ = 0.995). Data are presented as mean ± s.d. from three replicates. Model details are provided in Table S6.

Figure 6 shows a clear increasing dependence of F_1_/F_0_ value on transcribed RNA concentration, with the maximum response (F_1_/F_0_ = 5.40) recorded at 100 nM. The experimental *LOD* and *LOQ* values were determined to be 1.0 nM and 2.5 nM, respectively. Model-based estimates were slightly lower, calculated as 0.85 nM for *LOD* and 1.90 nM for *LOQ* (Table S8). The data were well-fitted using a four-parameter Hill model (R^2^ = 0.995), supporting the sensor’s reliable performance with longer transcribed RNA targets.

### 3.7. Adapted NASBA Strategy Enhances DNAzyme-Based Enterovirus Detection 

**Figure 7:**
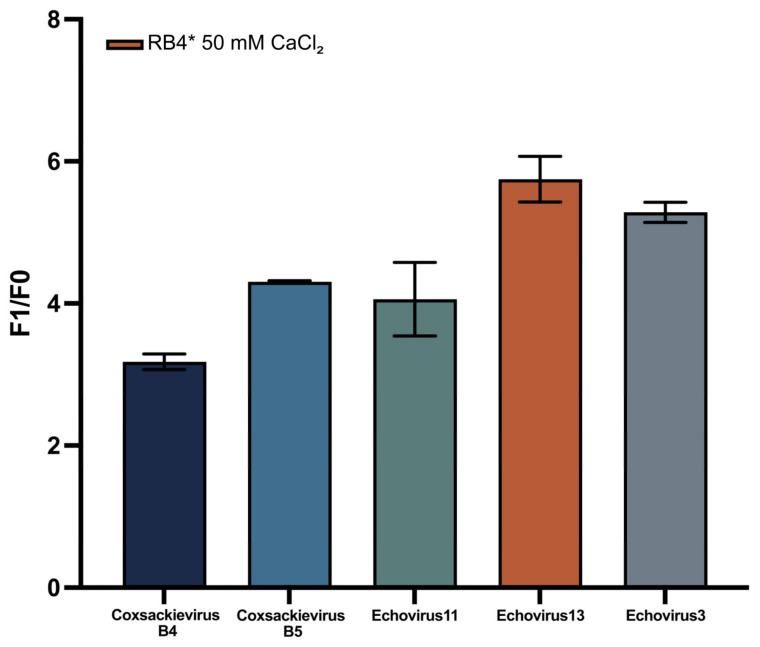
Signal ratio (F_1_/F_0_) of the DNAzyme-based biosensor in response to NASBA-amplified RNA from five enterovirus subtypes. Each subtype was tested under optimized assay conditions (Reaction Buffer 4* with 50 mM CaCl_2_). The system exhibited consistent signal generation across all groups, with the highest activation observed for Echovirus 3 and Echovirus 13. Data are shown as mean ± s.d. from three replicates.

The effectiveness of RNA-triggered DNAzyme activation is strongly influenced by the input RNA’s quality and accessibility. To ensure reliable signal generation, a modified NASBA-based RNA synthesis protocol was implemented using DNA templates derived from reverse transcription and PCR of viral cDNA. This approach was tested across five environmentally relevant enteroviruses (Figure 7), specifically: Coxsackievirus (B4, B5) and Echovirus (3, 11, 13). NASBA-derived RNA yielded reproducible fluorescent signals across all viral types. The biosensor system displayed excellent consistency, with coefficients of variation (CV) below 10% for all groups, indicating high reproducibility (Table S9). Coxsackievirus B5 exhibited the lowest variability (CV = 0.28%) and the narrowest signal range (0.0288), with 95% confidence interval (CI) of [4.284; 4.344], suggesting near-identical performance across replicates. The highest variation was observed with Echovirus 11 (CV = 7.68%), yet this still fell within acceptable reproducibility thresholds. Overall mean F_1_/F_0_ ratios ranged from 3.19 (Coxsackievirus B4) to 5.75 (Echovirus 3), reflecting subtype-specific amplification efficiencies. These results confirm the modified NASBA protocol’s ability to consistently generate functionally accessible RNA across diverse enteroviral strains. The high signal reproducibility, and well-defined confidence intervals, underscore the platform’s diagnostic robustness and highlight its suitability for multi-target detection in environmental samples.

## 4. Discussion

This study describes a DNAzyme-based biosensor designed for the detection of enteroviral RNA via a split-DNAzyme mechanism. The platform consists of two oligonucleotide strands (Dz1, Dz2) that hybridize to adjacent regions of the RNA target. Upon hybridization, the catalytic core is reconstituted, enabling cleavage of a fluorogenic hairpin substrate labeled with FAM at the 5’ end and BHQ1 at the 3’ end. Signal generation relies on highly precise, sequence-specific binding, ensuring nucleotide-level discrimination and minimizing off-target activation, even in complex sample matrices [29].

Additionally, the system demonstrated high specificity, producing a detectable signal only in the presence of the target enteroviral RNA, even when challenged with non-target synthetic viral RNAs. This feature is a major advantage, ensuring reliable performance in environmental matrices or complex samples where off-target interference is often problematic [19,30]. Validation using a panel of non-target viral RNAs, including norovirus, rotavirus A, human astrovirus, and others, showed no significant signal increase under non-target conditions. Statistical analysis confirmed strong target discrimination (*p* < 0.0001), supporting the sensor’s reliability in environmental applications.

To optimize the biosensor’s performance, various buffer systems (RB3*, RB4*) were evaluated with the addition of Triton X-100 and DMSO. These additives improved hybridization efficiency by preventing the formation of secondary structures, a critical limitation in DNAzyme-based systems. The resulting increases in signal strength and reproducibility (*p* = 0.0292) highlight the importance of optimizing reaction conditions for reliable performance [31,32]. Further enhancement of the catalytic environment was achieved by selecting Ca^2+^ over Mg^2+^ as the catalytic cofactor. Peak activity was observed at 48.5 mM Ca^2+^, attributed to its ability to stabilize the DNAzyme’s three-dimensional structure while minimizing non-specific conformational changes [32,33]. The optimized buffer formulation and Ca^2+^ concentration are easily reproducible in both laboratory and field settings, using globally available, low-cost, high-purity reagents (Tris-HCl, NaCl, CaCl_2_, and common additives). Moreover, the biosensor maintained stable signal output across a broad Ca^2+^ range (20–60 mM), demonstrating robustness to minor cofactor variations and ensuring consistent performance across diverse operational conditions.

In terms of analytical performance, the developed biosensor demonstrated high sensitivity to RNA concentration. It showed a linear response across the range of 0.5–100 nM, confirming the system’s capacity for RNA detection. Its low *LOD* and *LOQ* values highlight its potential for use with environmental samples containing low viral loads [27,34].

The biosensor also showed robust performance when tested with in vitro-transcribed RNA sequences containing secondary structural elements. These transcripts were synthesized from conserved regions of viral isolates to better mimic the complexity of naturally occurring viral RNA. Despite potential structural barriers that could hinder hybridization and catalytic cleavage, the system consistently generated detectable fluorescent signals. These results indicate that the DNAzyme configuration can tolerate such structural challenges in longer and folded RNA strands, consistent with previous literature [31].

The experimental *LOD* was determined to be 1.6 nM for synthetic RNA and 1.0 nM for in vitro-transcribed RNA. Model-based estimates using the Hill function yielded even lower values of 0.78 nM and 0.85 nM, respectively (Tables S6–S8). These sensitivities surpass those reported for several other split-DNAzyme systems. For example, Kirichenko et al. reported an *LOD* of 10 nM [15]. Certain platforms, such as the SPOT system, have achieved femtomolar-level detection for targets including miRNAs and SARS-CoV-2 RNA [35]. However, such ultra-sensitive architectures are typically unimolecular and allosteric, optimized for a single target. Such features increase design complexity and may limit adaptability. In this study, NASBA pre-amplification was applied to DNA templates obtained from preliminary reverse transcription and PCR steps, rather than directly to RNA extracts. While this approach differs from direct NASBA workflows, it still enables substantial amplification under isothermal conditions and demonstrates the compatibility of the DNAzyme module with NASBA products. This integration broadens the platform’s applicability to scenarios such as environmental surveillance and post-outbreak monitoring, offering a balanced combination of sensitivity, adaptability, and operational simplicity suitable for both field and multi-laboratory settings.

Beyond the *LOD*, the split 10–23 DNAzyme architecture provides notable design flexibility, as binding arms can be readily reconfigured for new RNA targets while keeping the catalytic core unchanged. The platform is protein-free in its detection step, can be multiplexed using different fluorophore–quencher pairs, and allows fine-tuning through buffer and cofactor adjustments. Combined with the use of portable fluorescence readers, these features enhance its suitability for rapid, field-deployable diagnostics.

Previous studies have shown that direct testing of unprocessed environmental matrices can result in reduced sensitivity due to inhibitory compounds [36]. In our trials, samples received after cell culture isolation, but before nucleic acid purification, were subjected directly to NASBA, omitting the RIBO-prep step. This approach failed to produce detectable cleavage signals. In contrast, the same samples yielded strong fluorescence after RNA purification. Consequently, all NASBA reactions in this study were performed on purified RNA templates.

In this study, a modified NASBA protocol was employed not for the conventional one-step isothermal amplification of native RNA, but rather as a two-stage strategy in which transcription and amplification were conceptually and procedurally separated. Traditionally, NASBA uses native RNA as the starting material. Primer-directed reverse transcription produces cDNA, and a second primer containing a T7 promoter enables in situ transcription of multiple RNA copies by T7 RNA polymerase at a constant temperature [34]. In contrast, our approach first generated sequence-verified double-stranded DNA from viral RNA via reverse transcription and T7-tagged PCR. This DNA served as a stable and accessible template for the NASBA reaction. In the subsequent NASBA step, the DNA template was amplified through transcription into large quantities of single-stranded RNA within 60 min at 41 °C, without the need for a separate T7 transcription kit. This adaptation was motivated by preliminary observations that direct NASBA on extracted viral RNA often yielded poor performance, likely due to strong secondary structures and limited primer accessibility. By decoupling the DNA generation and RNA amplification stages, the method ensured high transcription efficiency, consistent RNA yield, and sequence fidelity, even from partially degraded or low-titer starting materials. While this workflow deviates from classical NASBA as a one-step RNA-triggered amplification method, it retains its primary advantages (isothermal operation, minimal equipment requirements, rapid amplification) while overcoming challenges posed by structured viral RNA [20,26]. These findings highlight the chemical and operational flexibility of NASBA when repurposed for RNA synthesis from DNA templates, as well as its compatibility with downstream DNAzyme-based biosensing in field-deployable formats.

While the adapted NASBA protocol proved compatible with the DNAzyme detection module, its efficiency may decrease at extremely low target copy numbers, particularly with complex or inhibitor-rich matrices. In such cases, further optimization steps may be required to ensure robust amplification. These might include refined primer design, extended reaction times, improved enzyme formulations, or integration of upstream concentration/enrichment steps. Addressing these factors could broaden the method’s applicability to scenarios involving minimal viral loads, such as early-stage infections or post-outbreak environmental surveillance.

## 5. Conclusions

This study introduces a modular, DNAzyme-based biosensor platform that enables sequence-specific detection of enteroviral RNA under isothermal conditions. By using the structural programmability of a split 10–23 DNAzyme motif and optimizing both buffer composition and metal ion concentration, the system achieved strong catalytic activity, high signal reproducibility, and exceptional specificity, even in the presence of non-target viral RNAs. The system was validated using both synthetic and in vitro transcribed targets, demonstrating robust performance despite secondary structure complexity.

Beyond sensor design, a significant contribution of this work lies in the adaptation of NASBA chemistry. Rather than applying NASBA directly to native viral RNA, which often underperforms due to strong intramolecular structures, this platform repurposed NASBA as an RNA synthesis tool from DNA. Analytically, the biosensor exhibited a wide dynamic range and low detection limits, with consistent responses observed across five enteroviral strains isolated from real environmental sources. Statistical modeling and effect size analyses further confirmed the system’s robustness and diagnostic relevance. Compared to conventional molecular tools, DNAzyme-based sensing eliminates multi-enzyme dependency, reduces equipment burden, and supports on-site testing through portable fluorescence readouts.

Overall, the system presented here establishes a blueprint for amplification-coupled, DNAzyme-based sensing of pathogenic RNA. Its low-cost synthesis, high design flexibility, and strong signal fidelity position it as a powerful tool for decentralized viral surveillance. Future developments may focus on direct RNA amplification from raw samples, integration into field-deployable microdevices, and broadening of the detection panel. Such advances would enable real-world applications in outbreak monitoring, water safety testing, and point-of-care diagnostics. The aforementioned will hopefully bring programmable biosensing one step closer to accessible, real-time pathogen detection.

## Data Availability

Data is contained within the article or Supplementary Materials.

## Supplementary Materials

The following supporting information can be downloaded at: https://www.mdpi.com/article/10.3390/bios15090562/s1. Table S1: List of oligonucleotide sequences used; Table S2: Target RNA sequences; Table S3: Composition of tested reaction buffers; Table S4: Effect of reaction buffers on DNAzyme biosensor activity; Table S5: Calcium ion concentration-dependent activation of DNAzyme in optimized buffers; Table S6: Fluorescence response of DNAzyme biosensor to increasing concentrations of synthetic RNA; Table S7: Specific activation of DNAzyme biosensor by enteroviral RNA over non-target viral RNAs; Table S8: Sensitivity of DNAzyme biosensor to in vitro transcribed enteroviral RNA; Table S9: Detection of NASBA-amplified RNA from five enterovirus subtypes using the DNAzyme biosensor. All *LOD*/*LOQ* calculations are provided in detail alongside the Hill model parameters in Tables S6–S8.
