# Supplementary material for: An Isothermal Deoxyribozyme Sensor for Rapid Detection of Enteroviral RNA
Source: Biosensors (Basel). 2025 Aug 27;15(9):562. doi: 10.3390/bios15090562 (PMC12467329; doi:10.3390/bios15090562)
Supplement: Supplementary file 1 [file biosensors-15-00562-s001.zip › biosensors-3808186-supplementary.pdf]

Supporting Materials

An Isothermal Deoxyribozyme Sensor for Rapid Detection of Enteroviral RNA

Begüm Şaş [1,2,*], Anastasiia Dmitrievna Kirichenko [1], Marina Anatolyevna Kapitonova [1], Anna Vyacheslavovna Shabalina [1], Olga Ilyinichna Kanaeva [3], Tamer Mohammed El-Messery [2], Vladimir Georgievich Dedkov [1,4] and Anna Sergeevna Dolgova [1]

1 Laboratory of Pathogen Molecular Genetics, Saint Petersburg Pasteur Institute, 197101 St. Petersburg, Russia; anastasiiakirichenko97@gmail.com (A.D.K.); kapitonova.marin@gmail.com (M.A.K.); shabalina.anna.v@gmail.com (A.V.S.); vgdedkov@yandex.ru (V.G.D.); annadolgova@inbox.ru (A.S.D.)
2 Faculty of Biotechnologies, ITMO University, 191002 St. Petersburg, Russia; tmelmessery@yahoo.com
3 Laboratory of Etiology and Control of Viral Infections, Saint Petersburg Pasteur Institute, 197101 St. Petersburg, Russia; ol.kanaeva@gmail.com
4 Martsinovsky Institute of Medical Parasitology, Tropical and Vector Borne Diseases, Sechenov First Moscow State Medical University (Sechenov University), 119048 Moscow, Russia
* Correspondence: begum.sas99@gmail.com

Table S1. List of oligonucleotide sequences used in this work.

Name	Sequence (5`→3`)
EV Forward	TATTACGACTCACTATATGGGcccagtgtagatcaggtcgatgaag
EV Reverse	gattagccgccgcattcaggggcc
Assembly 1	CCCCAGTGTAGATCAGGTCGATGAGTCACCGCGTTCCCCACGGGTGACCGTGGC
Assembly 2	AGCGTCCCATGGGTTGCCCCATGGGCAGGCCGCCAACGCAGCCACCGCCAC-GGTCACCCG
Assembly 3	CAACCCATGGGACGCTTCAATACTGACATGGTGCGAAGAGTCTATTGAGCTAATTGGTAG
Assembly 4	CAGTTAGGATTAGCCGCATTCAGGGGCCGGAGGACTACCAATTAGCTCAATAGACTC
FAM	AAG GTT(FAM) TCC TCg uCC CTG GGC A(BHQ1)

Here: A, G, T, C – DNA nucleobases, a, g, u, c – RNA nucleobases;
modifications:
[FAM] – Fluorescein (3′,6′-dihydroxyspiro[isobenzofuran-1(3H),9′-[9H]xanthen]-3-one),
[BHQ-1] - Black Hole Quencher 1 (2-[N-(2-hydroxyethyl)-4-[[2-methoxy-5-methyl-4-[(4-methyl-2-nitrophenyl)di-azenyl]phenyl]diazenyl]anilino]ethanol).

Table S2. List of target sequences used in this work.

Name	Sequence (5`→3`)
EV Dz1	agacucuucgACAACGAGAGGAAACCTT
EV Dz2	TGCCCAGGGAGGCTAGCTcaccauguca
Enterovirus	uucaatacugacauggugcgaagagucuauugagcu
Norovirus GII	auggugagauccaucgaggaugcaguacuagaccucgaggcaauugaaaaca
Rotavirus A	taaatggtctgaagtttctttcaaagcagcaaattatcagtacacctatttacgtga
Human Astrovirus	aaacagggugucacaggaccaaaaccugcaaaugccagagagccacugcaaca
Human Adenovirus 41	gaaacaacagccuuuguaacaacuguacugcuacuacuuuaacuccuuacaccuc
Sapovirus	ugaauuugacaccgaucgugaugccaaguuuguugaacaaaugauugc
Hepatitis A Virus	gagcuguaggagucuaaauuggggacgcagauguuugggacgucaccuu

Table S3. Composition of Reaction Buffers.

Reaction Buffers	Composition
RB1	50 mM Na-HEPES, 25 mM EDTA, pH 7.5
RB1*	50 mM Na-HEPES, 25 mM EDTA, 0.06% Triton X-100, 0.05% DMSO, pH 7.5
RB2	50 mM Tris-HCl, pH 7.5
RB2*	50 mM Tris-HCl, 0.06% Triton X-100, 0.05% DMSO, pH 7.5
RB3	100 mM HEPES, 300 mM KCl, pH 7.5
RB3*	100 mM HEPES, 300 mM KCl, 0.06% Triton X-100, 0.05% DMSO, pH 7.5
RB4	140 mM NaCl, 5 mM KCl, 50 mM HEPES, pH 7.4
RB4*	140 mM NaCl, 5 mM KCl, 50 mM HEPES, 0.06% Triton X-100, 0.05% DMSO, pH 7.4

Table S4. Effect of reaction buffers on DNAzyme biosensor activity.

Enterovirus 5, 2.5 mM Ca2+	F1	F0	F1	F0	F1	F0	F1/F0	F1/F0	F1/F0	Average
Buffer 1	3637,4	712,4	3516,5	772,1	2919,6	806,1	4,088172499	4,554461857	3,621883141	4,088172499
Buffer 1*	4414,9	1118,6	3191,6	877,8	4567,9	924,4	3,946808511	3,635907952	3,791358231	3,791358231
Buffer 2	2174,8	603,5	3156,3	636,7	2665,55	530,5	3,603645402	4,957279724	4,280462563	4,280462563
Buffer 2*	3997,1	850,1	3428,7	827,7	3506	875,1	4,701917421	4,142442914	4,006399269	4,283586535
Buffer 3	2225,5	657,9	2447,6	515,3	2285,8	506,3	3,382732938	4,749854454	4,514714596	4,215767329
Buffer 3*	**3620,7**	**629,7**	**3117,7**	**670,4**	**3054,3**	**609**	**5,749880896**	**5,382575916**	**5,015270936**	**5,382575916**
Buffer 4	3825	1043,3	4242,3	907,6	4276,1	844,1	3,666251318	4,674195681	4,170223499	4,170223499
Buffer 4*	**3633,9**	**760,5**	**3861,1**	**848,8**	**3902,1**	**843**	**4,778303748**	**4,548892554**	**4,628825623**	**4,652007308**

Table S5. Calcium ion concentration-dependent activation of DNAzyme in optimized buffers.

Reaction Buffer 4*

Buffer 4*, CA, 41 Degree	F1	F0	F1	F0	F1	F0
100 mM	3680,8	862,6	3611	800,7	4017,6	835,9
75 mM	3857,3	742,3	4140,3	712,8	3933,9	852,9
50 mM	4442,7	692	4071,7	710,1	3245,8	701,05
25 mM	3870,2	801,4	4009,7	733,5	3812,8	692,6
10 mM	3637,9	963,9	3690,3	651,2	3676,4	617,4
5 mM	4123,1	626,1	3530,8	820,1	3530,8	885,8

Average F = 0

Buffer 4*, CA, 41 Degree	F0	F0	F0	Average
100 mM	862,6	800,7	835,9	833,0666667
75 mM	742,3	712,8	852,9	769,3333333
50 mM	692	710,1	701,05	701,05
25 mM	801,4	733,5	692,6	742,5
10 mM	963,9	651,2	617,4	744,1666667
5 mM	626,1	820,1	885,8	777,3333333

Buffer 4*, CA, 41 Degree	F1/F0	F1/F0	F1/F0	Average
100 mM	4,41837388	4,334587068	4,822663252	4,525208067
75 mM	5,01382149	5,381672444	5,113388215	5,169627383
50 mM	6,420086705	5,733981129	4,629912274	5,594660036
25 mM	5,212390572	5,40026936	5,135084175	5,249248036
10 mM	4,888555431	4,958969765	4,940291153	4,929272116

5 mM	5,30415952	4,860077187	4,54219554	4,902144082

Polynomial Regression Model for Buffer 4* (CA, 41 °C)

A third-degree polynomial regression was applied to model the relationship between $CaCl_2$ concentration (x in mM) and the fluorescence response (F_1/F_0). The resulting model is:

$$F_1/F_0 = 4.6873 + 0.0328x - 0.00032x^2 - 2.61 \times 10^{-7}x^3 \quad (1)$$

The coefficient of determination (R^2) for this fit is:

$$R^2 = 0.9634$$

Reaction Buffer 3*

Buffer 3*, CA, 41 Degree	F1	F0	F1	F0	F1	F0
100 mM	**3808,9**	**636,9**	**3410,4**	**661,9**	**3408,7**	**642,9**
75 mM	3536,6	718,4	3650,8	746	3873,7	732,2
50 mM	3225,8	755,9	3323,9	686,2	3302,7	612,4
25 mM	3374,6	655,8	3295,6	766,9	3359	592,3
10 mM	3319,3	611,4	3427,9	673,8	3368,5	770,8
5 mM	3019	766,5	2763,5	668,3	2510,2	774,6

Average = F0

Buffer 3*, CA, 41 Degree	F0	F0	F0	Average
100 mM	**636,9**	**661,9**	**642,9**	647,2333333
75 mM	718,4	746	746	736,8
50 mM	755,9	686,2	612,4	684,8333333
25 mM	655,8	766,9	592,3	671,6666667
10 mM	611,4	673,8	770,8	685,3333333
5 mM	766,5	668,3	774,6	736,4666667

Buffer 3*, CA, 41 Degree	F1/F0	F1/F0	F1/F0	Average
100 mM	**5,88489468**	**5,269197095**	**5,266570531**	**5,473554102**
75 mM	4,830101065	4,98606938	5,2904944	5,035554949
50 mM	4,710343149	4,853589681	4,822633244	4,795522025
25 mM	5,024218362	4,906600496	5,000992556	4,977270471
10 mM	4,843336576	5,001799611	4,915126459	4,920087549
5 mM	4,099302978	3,752376211	3,40843668	3,753371956

Polynomial Regression Model for Buffer 3* (CA, 41 °C)

A third-degree polynomial regression was applied to model the relationship between $CaCl_2$ concentration (x in mM) and the fluorescence response (F_1/F_0). The resulting model is:

$$\frac{F_1}{F_0} = 3.7168 + 0.0898x - 0.00183x^2 + 1.11 \times 10^{-5}x^3 \quad (2)$$

The coefficient of determination (R^2) for this fit is:

$$R^2 = 0.7260$$

Table S6. Fluorescence response of DNAzyme biosensor to increasing concentrations of synthetic RNA.

3-Parameter Hill Function

$$y = A + \frac{D - A}{1 + \left(\frac{C}{x}\right)^B} \quad \text{where } A = 1.00$$

Parameter	Description	Value
A	Minimum signal (fixed)	1.000
D	Maximum response	**5.387**

Parameter	Description	Value
C	EC_{50} (half-max. conc.)	**3.280**
B	Hill slope coefficient	**1.043**

$$R^2 = 1 - \frac{\sum(y_i - \hat{y}_i)^2}{\sum(y_i - \bar{y})^2}$$

LOD and LOQ Estimation Based on Blank Signal

LOD $= 3.3 \cdot \sigma/S$ **LOQ** $= 10 \cdot \sigma/S$

Mean blank signal: $\bar{F}_0 = 769.56$ **Standard deviation:** $\sigma_{F_0} = 80.27$

$LOD_{ratio} = 1010.35769.56 = 1.313$ $LOQ_{ratio} = 1572.21769.56 = 2.043$

To estimate corresponding RNA concentrations, the inverse of the Hill function was applied:

$$x = C \cdot \left(\left(\frac{D - A}{y - A}\right) - 1\right)^{-1/B}$$

$LOD_{conc} = 0.280\,nM$ $LOQ_{conc} = 1.074\,nM$

Experimental Blank

Mean blank signal: $\bar{F}_0 = 769.56$
Standard deviation: $\sigma_{F_0} = 80.27$

$$\text{LOD}_{\text{signal}} = \bar{F}_0 + 3\sigma = 769.56 + 3 \cdot 80.27 = \mathbf{1010.35}$$
$$\text{LOQ}_{\text{signal}} = \bar{F}_0 + 10\sigma = 769.56 + 10 \cdot 80.27 = \mathbf{1572.21}$$

LOD detection point: 0.5 nM $\rightarrow$ $F_1 = 1127.8 > 1010.35$ $\rightarrow$ **meets LOD**
LOQ detection point: 2.5 nM $\rightarrow$ $F_1 = 2025.0 > 1572.21$ $\rightarrow$ **meets LOQ**

Table S7. Specific activation of DNAzyme biosensor by enteroviral RNA over non-target viral RNAs.

Grup	Ratio F_1/F_0	SD	CV (%)
Enterovirus	4,6222	0,4639	10,04
Hepatitis A	1,0574	0,0404	3,82
Astrovirus	0,9514	0,1903	20,00
Norovirus	1,0489	0,1072	10,22
Sapovirus	0,9907	0,1004	10,13
Rotavirus A	1,0462	0,1188	11,35
Adenovirus	0,9115	0,0727	7,98
NTC	1,0000	0,0000	0,00

Between-group sum of squares (SSB): 34,4784, Within-group sum of squares (SSW): 0,5878
Degrees of freedom: $df_1 = 7$, $df_2 = 16$, F-value: ~423, p-value: < 0,0001

Table S8. Sensitivity of DNAzyme biosensor to in vitro transcribed enteroviral RNA.

The Hill function used to fit the sensor response is:

$$y = A + \frac{D - A}{1 + \left(\frac{C}{x}\right)^B} \quad \text{where} \quad A = 1.00$$

Parameter	Description	Value
A	Minimum signal (fixed)	1.000
D	Maximum response	5.392
C	EC_{50} (half-max. conc.)	3.675
B	Hill slope coefficient	1.08

$$R^2 = 1 - \frac{\sum(y - \hat{y})^2}{\sum(y - \bar{y})^2} \quad \Rightarrow \quad R^2 = 0.995$$

Blank Signal Statistics:

$$\begin{aligned} &\text{Mean blank signal: } \bar{F}_0 = 855.1 \\ &\text{Standard deviation: } \sigma = 46.12 \\ &\text{LOD}_{\text{signal}} = \bar{F}_0 + 3\sigma = 993.47 \\ &\text{LOQ}_{\text{signal}} = \bar{F}_0 + 10\sigma = 1316.35 \end{aligned}$$

To estimate corresponding RNA concentrations, the inverse Hill function was applied:

$$x = C\left(\left(\frac{D-A}{y-A} - 1\right)^{1/B}\right)$$

$$\text{LOD}_{\text{conc}} = 3.675\left(\left(\frac{5.392-1}{1.162-1} - 1\right)^{1/1.08}\right) \approx 0.85 \text{ nM}$$

$$\text{LOQ}_{\text{conc}} = 3.675\left(\left(\frac{5.392-1}{1.54-1} - 1\right)^{1/1.08}\right) \approx 1.90 \text{ nM}$$

Validation:

$$\begin{aligned} &\text{At 1.0 nM, mean } F_1 = 1003.2 \Rightarrow \text{exceeds LOD}_{\text{signal}} \\ &\text{At 2.5 nM, mean } F_1 = 1448.9 \Rightarrow \text{exceeds LOQ}_{\text{signal}} \\ &\min(x) \text{ such that } F_1 > \text{LOD}_{\text{signal}} = 993.47 \Rightarrow 1.0 \text{ nM} \\ &\min(x) \text{ such that } F_1 > \text{LOQ}_{\text{signal}} = 1316.35 \Rightarrow 2.5 \text{ nM} \end{aligned}$$

Table S9. Detection of NASBA-amplified RNA from five enterovirus subtypes using the DNAzyme biosensor.

Virus Type	Mean (F_1/F_0)	SD	CV (%)	Range	CI Lower	CI Upper
Coxsackievirus B4	3.187	89	2.79	0.2146	2.965	3.409
Coxsackievirus B5	4.3139	12	0.28	0.0288	4.284	4.344
Echovirus 11	4.7417	364	7.68	0.8889	3.838	5.645
Echovirus 13	5.2902	122	2.31	0.2858	4.987	5.593
Echovirus 3	5.7525	257	4.47	0.6443	5.115	6.39

CV (%)	Interpretation
< 10%	High repeatability / consistency
10–30%	Acceptable
> 30%	Low reliability — method should be re-evaluated
